# Seahorses in focus: local ecological knowledge of seahorse-watching operators in a tropical estuary

**DOI:** 10.1186/s13002-016-0125-8

**Published:** 2016-11-08

**Authors:** Maria L. F. Ternes, Leopoldo C. Gerhardinger, Alexandre Schiavetti

**Affiliations:** 1 Programa de Pós Graduação em Zoologia, Universidade Estadual de Santa Cruz, Bahia, Brazil; 2 Universidade Regional de Joinville, São Francisco do Sul, Santa Catarina Brazil; 3 Departamento de ciências Agrárias e Ambientais, Universidade Estadual de Santa Cruz, Bahia, Brazil

**Keywords:** Syngnathidae, *Hippocampus reidi*, Ethnobiology, Mangrove, Brazil, Pernambuco, *Jangadeiros*, Conservation, Management, Tourism

## Abstract

**Background:**

Seahorses are endangered teleost fishes under increasing human pressures worldwide. In Brazil, marine conservationists and policy-makers are thus often skeptical about the viability of sustainable human-seahorse interactions. This study focuses on local ecological knowledge on seahorses and the implications of their non-lethal touristic use by a coastal community in northeastern Brazil. Community-based seahorse-watching activities have been carried out in Maracaípe village since 1999, but remained uninvestigated until the present study. Our goal is to provide ethnoecological understanding on this non-extractive use to support seahorse conservation and management.

**Methods:**

We interviewed 32 informants through semi-structured questionnaires to assess their socioeconomic profile, their knowledge on seahorse natural history traits, human uses, threats and abundance trends.

**Results:**

Seahorse-watching has high socioeconomic relevance, being the primary income source for all respondents. Interviewees elicited a body of knowledge on seahorse biology largely consistent with up-to-date research literature. Most informants (65.5 %) perceived no change in seahorse abundance. Their empirical knowledge often surpassed scientific reports, i.e. through remarks on trophic ecology; reproductive aspects, such as, behavior and breeding season; spatial and temporal distribution, suggesting seahorse migration related to environmental parameters.

**Conclusions:**

Seahorse-watching operators were aware of seahorse biological and ecological aspects. Despite the gaps remaining on biological data about certain seahorse traits, the respondents provided reliable information on all questions, adding ethnoecological remarks not yet assessed by conventional scientific surveys. We provide novel ethnobiological insight on non-extractive modes of human-seahorse interaction, eliciting environmental policies to integrate seahorse conservation with local ecological knowledge and innovative ideas for seahorse sustainable use. Our study resonates with calls for more active engagement with communities and their local ecologies if marine conservation and development are to be reconciled.

## Background

Seahorses are teleost fishes, belong to Syngnathidae within the genera *Hippocampus* and are currently represented by 41 species distributed worldwide [1]. Some life story traits make seahorses vulnerable to human pressures, such as: low mobility, small home range, low population density, a predominantly monogamous behavior, low fertility and a long period of parental care [2]. Although they are not used for human food, seahorses have significant commercial value around the world [3]. These iconic fishes are exploited by extractive activities such as fishing (intentional and incidental) for ornamental trade [4] and for dried trade used in traditional medicine in Asian countries [5–7]. Every year, millions of seahorses are caught to supply a multimillion market involving 93 countries, causing overexploitation at alarming rates [5]. Besides human use, seahorse populations are globally threatened by habitat loss [6].

Seahorse identification is controversial and complex, mainly challenged by morphological and phenotypic plasticity [1]. In Brazil, three species have been described: *Hippocampus reidi*, *H. erectus* and *H. patagonicus* [1]. *Hippocampus reidi* is the most abundant species along the Brazilian coast [8] but it has suffered from collection for ornamental trade, with Brazil being the leading exporter in Latin America [4].

*Hippocampus reidi* was listed globally as “vulnerable” in the red list of endangered species of the International Union for Conservation of Nature in 1996 [9]. However, in 2003, it was reclassified as “data deficient”, like 26 other seahorse species, highlighting the urgent need for data to define its status. In addition, the genus *Hippocampus* is cited in Appendix II of the Convention of International Trade of Endangered Species of Fauna and Flora [10], which lists species of this genus as threatened by overexploitation. In the Brazilian list of endangered species [11], *H. reidi* was listed as “vulnerable” due to populational decline, overexploitation, lack of fishery landing data and habitat loss.

**Fig. 1:**
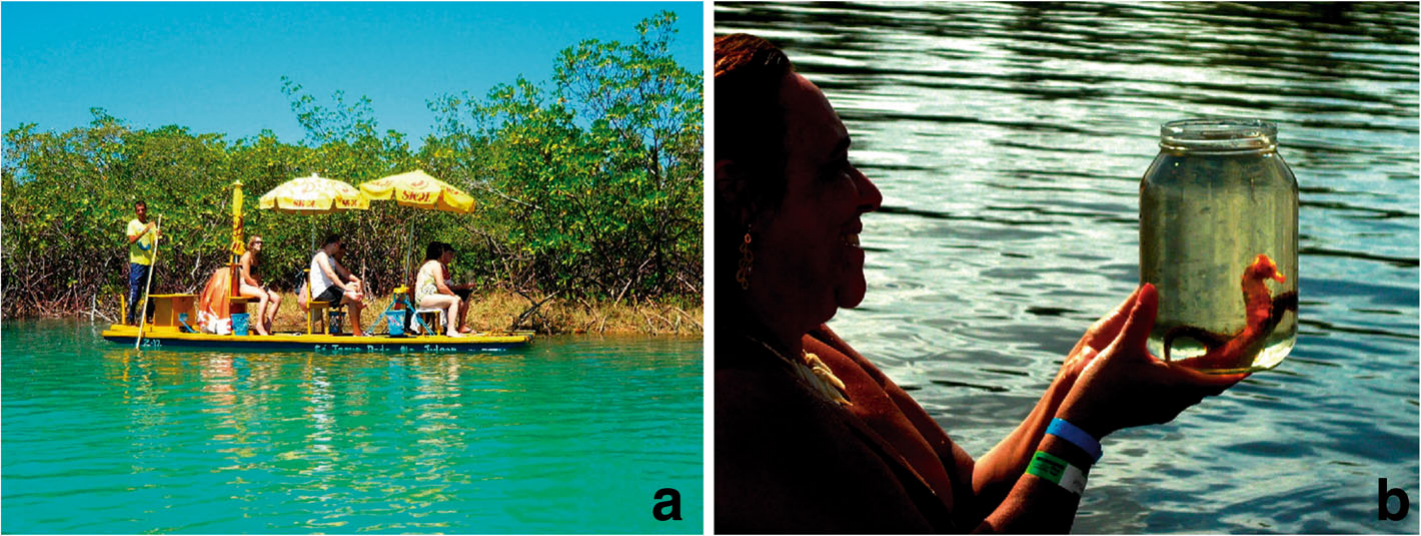
**a** The traditional *jangada,* used by seahorse-watching stakeholders in Maracaípe*;* **b** a visitor observing seahorses on a glass container. Credits: MLF Ternes. (Images published under previous consent of the participants)

In Maracaípe estuary (state of Pernambuco, northeastern Brazil), local villagers known as *jangadeiros* (rafters) interact with *H. reidi* in a non-lethal mode. By navigating a *jangada* (traditional wooden raft boat, Fig. 1a), they guide tourists to watch seahorses in a mangrove area, where *jangadeiros* free dive to collect the specimens, which are then held in glass containers for the tourists to observe (Fig. 1b). *Jangadeiros* provide information about the seahorses and release them back to their natural habitat. This activity has been taking place in Maracaípe for more than a decade (since 1999 [12]), but remained uninvestigated until the present study. To date, despite local arguments for the sustainable nature of this alternative livelihood, some seahorse conservationists and policy-makers remain skeptical about such novel human-seahorse modes of interaction.

For instance, in 2011, the Brazilian government gathered scientists to elaborate the “Proposal of management plan for the sustainable use of seahorses” [13]. The proposal focuses mainly on extractive use through fishing, lacking scientific data about the non-extractive users who depend on seahorses as a touristic resource in Maracaípe. Even though, the proposal regards the touristic use a threat, arguing it is an unordered activity where stakeholders have no knowledge of seahorse biology and pass on unreliable information to tourists. The technical report therefore recommends ethnoecological and socioeconomic studies to provide data on resource users to support seahorse conservation and management [13].

The ethnoecological approach investigates the local ecological knowledge (LEK) held by humans about the environment and natural resources, especially those necessary for subsistence [14]. Such knowledge is particularly important to understand human patterns of resource use for management and long-term sustainability, especially in places where scientific knowledge about local human and ecological processes of the seascape is often unknown [15, 16]. Thus, research on the human use of seahorses can fill in gaps of biological and ecological information on these fishes and is often considered to be necessary to subsidize their management and conservation [2, 3, 13].

Our research investigates an outstanding case of community spontaneous self-organization, located on a far side of the increased openness for participation in research spectrum [17], an intriguing case of self-governing mode of community seahorse conservation (see [18]). Our analysis of the socioeconomic profiles of *jangadeiros* and their knowledge on biological and ecological aspects of seahorses may thus elicit regulatory gaps and innovative ideas for seahorse conservation plans in Brazil.

## Methods

### Study site

Maracaípe is a village located in the municipality of Ipojuca, state of Pernambuco, northeastern Brazil (8°31′00″ S, 34°59′30″ W). It comprises a sandy beach about 3.8 km long, with mangrove and sandstone reefs at its southern portion, known as Pontal, where the Maracaípe river estuary meets the sea. Maracaípe is located near Recife (70 km), the most populous city of Pernambuco, neighboring an important site of beach tourism, Porto de Galinhas.

Seahorse-watching is conducted in a mangrove area on the estuarine portion of the Maracaípe River. The vegetation is mostly composed of *Rhizophora mangle*, *Laguncularia racemosa* and *Avicennia schaueriana*. The depth ranges from 0.25 m at low tide to 2.7 m at high tide [19]. Water temperature varies between 26 and 29 °C in austral winter (June to September) and between 27.9 and 32 °C in austral summer (December to March) [20].

### Surveyed community

The informants interviewed are known as “*jangadeiros*”, because they handle a seven meter long wooden raft boat called “*Jangada”*, a traditional fishing boat used in northeastern Brazil coastal communities. *Jangadas* raft silently and smoothly by human propulsion in the shallow waters of Maracaípe’s mangrove. Each *jangadeiro* uses a five meter wooden pole to touch the ground and by pushing it, the boat moves ahead.

*Jangadas* play a historical role in artisanal fishing, but the low profitability and precarious work conditions combined with depletion of fish stocks, led *jangadeiros* to engage in other occupations [12]. With increased opportunities in tourism, *jangadas* have been used in touristic activities in Maracaípe, as well as in other coastal localities in northeastern Brazil. The *jangadeiros* of Maracaípe have been organized into an association since 1999, when seahorse-watching had been implemented by their own initiative. It encompassed 38 members in 2012 (survey period) and 40 members in January 2016.

### Data collection and analysis

Interviews were approved by the Ethics Committee of Universidade Estadual de Santa Cruz (protocol: 08269112.0.0000.5526) and conducted between April 2012 and April 2013. Ethnoecological data were collected respecting local cultural identity and establishing a mutual relationship of trust between researcher and informants [21]. All *jangadeiros* were invited to participate in the study. After previous consent, semi-structured interviews were individually applied. The questionnaire addressed questions on (1) Socioeconomic profile: age, level of education, experience, income source; (2) Ethnotaxonomy: species name, richness, species description; (3) Distribution: habitat, environmental parameters, holdfast preferences; (4) Trophic ecology: diet, feeding behavior, predators; (5) Reproductive aspects: sexual dimorphism, behavior, brood size, survival rate, reproductive period; (6) Human uses of seahorses, and (7) Population abundance trends and threats to seahorse conservation.

Descriptive statistics were used to analyze responses from interviewees and results were presented as counts and percentages. Data were analyzed qualitatively under an emic–etic perspective [22] to contrast community knowledge and intentions with academic scholarship. For comparative purposes, we used a table of “consensual responses” (when more than 50 % of the informants shared a similar response to a given question). A level of response fidelity (LF) was calculated with the formula LF = (CI/TI) × 100, where CI = number of informants who cited the consensual response (most frequently quoted response), TI = Total number of informants (n). The differences between seahorse abundance trends and *jangadeiros’* age were verified using Kruskal-Wallis one-way analysis of variance since data were nonparametric.

## Results

### Socioeconomic profile

We interviewed 32 informants encompassing 84 % of the total 38 *jangadeiros* operating the seahorse-watching. Five *jangadeiros* declined to participate in the survey because of a past negative experience with another researcher. All interviewees were male, with ages ranging from 21 to 59 years (average = 35 years) and seahorse-watching experience ranging from seven months to 13 years (average = 9 years) (Table 1). Sixty-six percent of the *jangadeiros* had a low education level, below middle school. Former occupations were mostly fishing and crustacean/mollusk extraction (62.5 %). Seahorse-watching was the only income source for 84 % of the *jangadeiros*, while 19 % had complementary activities.

**Table 1:** *Jangadeiros* socioeconomic profile (*n* = 32)

	No. of informants	Frequency (%)
Age class		
21 – 30	8	25
31- 40	18	56
41 – 50	6	19
51 – 59	1	3
Education level (years of schooling)		
Illiterate	5	16
Elementary school incomplete (2 years)	2	6
Elementary school (5 years)	14	44
Middle school (9 years)	8	24
High school (12 years)	4	12.5
Experience in seahorse-watching (years)		
< 1	1	3
1 – 7	14	44
8 – 13	18	56
Former occupation		
Fishing	20	62.5
Others	15	47
Income source		
Only seahorse-watching	27	84
Complementary activities	6	19

Seahorse-watching actors are locally organized in an Association of *Jangadeiros* that self-regulates their activity in the Maracaípe estuary and seems engaged and interested in developing an approach for sustainable use of seahorses. They have established self-enforced norms to regulate seahorse-watching, as well as their interaction with seahorses and the surrounding environment, such as seahorse handling procedures and organization of mangrove cleanup days.

### Local ecological knowledge on seahorses

#### Ethnotaxonomy

Up to four ethnospecies were mentioned for the Maracaípe estuary and adjacent region (Table 2). Most informants (62.5 %) cited only one ethnospecies, while 31 % cited two, and 9 % cited three or more. One *jangadeiro* classified seahorses according to color, claiming that “dark” individuals (brown and black) were a native mangrove species, while the “colored” ones (other tonalities) belonged to different species native from the sea.

**Table 2:** Seahorse ethnospecies description, according to 32 informants

Ethnospecies names	Description	Citations (%)	Habitat
*Cavalo-marinho do focinho longo, Cavalo-marinho do manguezal, Reidi* (Longsnout, mangrove-seahorse or “Reidi”)	Long snout	32 (100 %)	Mangrove and reefs
*Cavalo-marinho do focinho curto, Erecto* (Shortsnout or “Erecto”)	Short snout. Less common than the Longsnout seahorse.	5 (16 %)	Mangrove and reefs
*Cavalo-marinho Folha* (Leafy-seahorse)	Seahorse head and leafy shaped body	1 (3 %)	Rocks near the river mouth
*Cavalo-marinho-rei,* *Cavalo-marinho-verdadeiro* (King-seahorse or True-seahorse)	Crown on top of head like a “moose horn”. Body more robust than the common mangrove seahorse. Has strictly marine habits. Reported as a bycatch of bottom-trawlers operating in nearby fishing communities.	6 (19 %)	Occasionally found in trawls at ~30 m. Also named true-seahorse because inhabits the sea bottom rather than mangroves.

### Distribution

According to all interviewees, seahorse inhabits both mangroves and reefs. Regarding depth distribution, 81 % did not mention a specific preferred depth for seahorse occurrence in the Maracaípe mangrove. The respondents were nearly unanimous (97 %) in stating that seahorses prefer backwater areas, more protected from the action of currents. The occurrence of seahorses was related to holdfasts. All *jangadeiros* mentioned that the mangrove roots of *L. racemosa*, *R. mangle,* and *A. schauerianna* were the main anchoring points; followed by algae (*Sargassum* spp., 28 %); submerged mangrove branches (25 %); corals and rocks (28 %); seagrass (3 %); and artificial structures as anchors and lines (3 %).

**Fig. 2:**
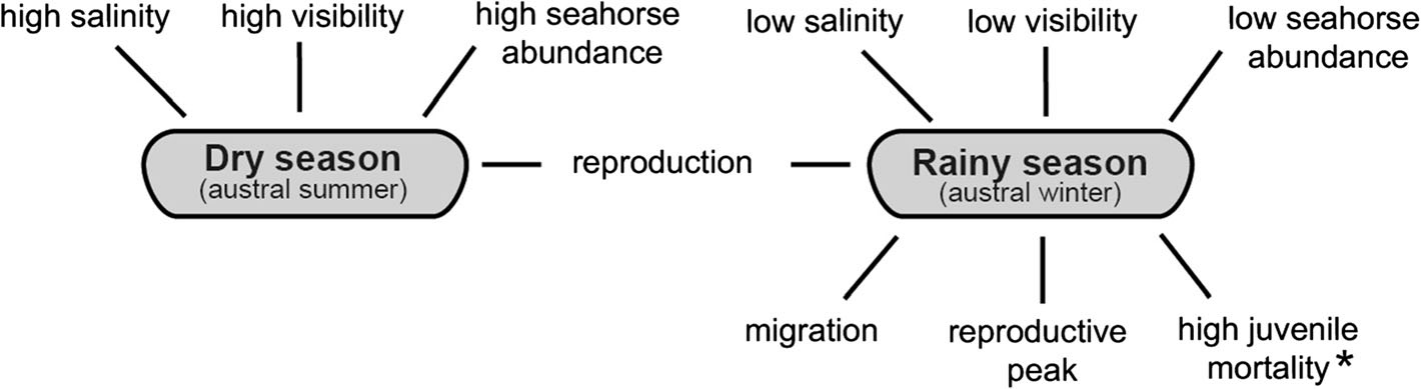
Summary of events that occur in the dry and rainy seasons in the Maracaípe estuary, according to the knowledge of *jangadeiros*. *Provided through biological data [23]

All informants reported that seahorses prefer areas with higher levels of salinity and visibility. Seasons were mentioned as determinants for seahorse abundance (Fig. 2), as it was always difficult to find them in winter, which locally corresponds to the rainy season, from May to September. According to all *jangadeiros,* the limiting factors for seahorse-watching activities during this period were poor visibility and scarcity of individuals. All the interviewees reported that in the rainy season the freshwater input decreases the salinity levels inside the estuary. Additionally, 97 % explained that seahorses are absent during this period because they move to the sea seeking saline waters, and are also removed out of the mangrove by the strong rain flood and currents. Figure 2 summarizes all the events mentioned to occur during the dry and rainy seasons. A single informant claimed that seahorses still remain in the mangrove during rainy season, but are not found because of poor visibility, as the water turbidity increases.

### Trophic ecology

**Fig. 3:**
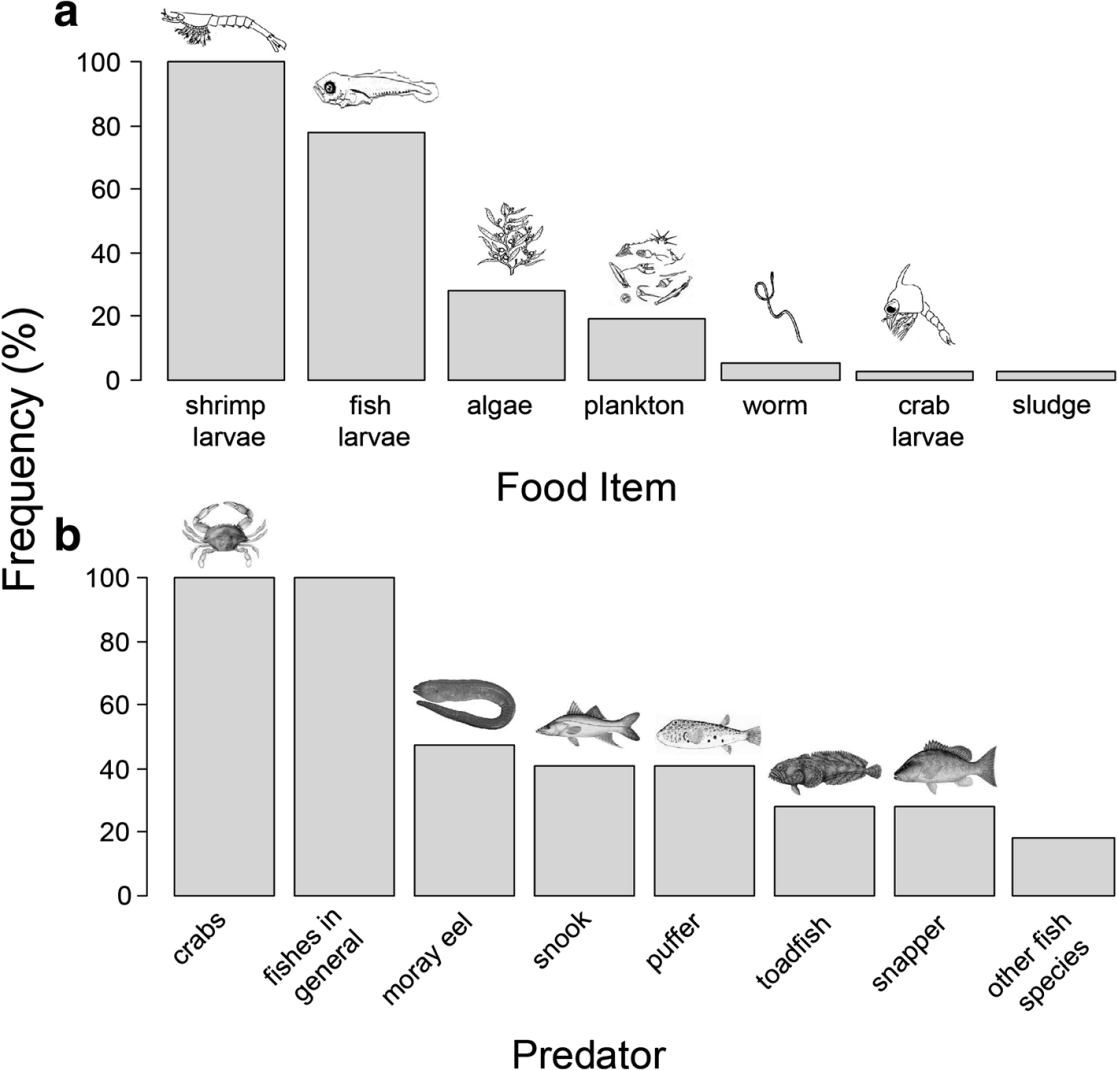
**a** Seahorse food items; and **b** predators according to *jangadeiros*’ knowledge in the Maracaípe estuary (*n* = 32). Family of predators: Morey eel (Muraenidae), snook (Centropomidae), puffer (Tetraodontidae), toadfish (Batrachoididae), *Lutjanus* sp. (Lutjanidae)

The main seahorse food items quoted were shrimp larvae (100 %) and fish larvae (78 %), among algae, plankton, worms, crab larvae and others (sediment, sludge) (Fig. 3a). The informants additionally described that “*seahorses feed on anything that fits into their mouth*”. Regarding feeding behavior, 97 % mentioned that seahorses rapidly strike the prey, sucking it. Some respondents additionally described that seahorses usually stay still, waiting for the prey to pass (25 %), and during the strike, a snapping sound is produced (9 %). Seahorses can also detach from anchoring points and move around to inspect the area and forage, especially in low tide or intertidal periods, when the currents are weaker (9 %).

Crabs (*Callinectes* spp.) and fishes in general were mentioned as seahorse predators by all *jangadeiros*, besides specifying certain estuarine fish species (Fig. 3b). According to them, such predation was experienced directly during diving, or, in the case of crabs, also implied by predation marks, amputation and scars left on the bodies of seahorses.

### Reproductive aspects

All the informants explained that males have a ventral brood pouch, being the ones who “get pregnant”. Fifty-nine percent had already seen the seahorses’ birth in the collection containers, while displaying them to tourists. Birth behavior was described as ventral contractions, releasing a “cloud of newborns”, miniature of adults. Brood size was said to range from a minimum of 100 to 1300 newborns, although 91 % of the informants quoted 500 to 1000 offsprings per pregnancy.

Most interviewees (91 %) quoted survival rates of up to 15 % and those who could not give a rate (6 %) admitted that only few newborns survive, because of the high predation on early life stage. Only one respondent (a beginner with seven months of experience in seahorse-watching) quoted higher rates, up to 50 %. According to all *jangadeiros*, *H. reidi* reproduction occurs throughout the year. Some respondents (25 %) detailed that it intensifies during the rainy season (winter) (Fig. 2) when they observe an increase of egg-bearing males: “*In winter, all males are pregnant all the time, we can hardly find one who is not*” (39-year-old informant).

### Human uses of seahorses

The current use of seahorses reported by Maracaípe *jangadeiros* was restricted to its non-consumptive exploitation as a touristic resource. The most popular use in the past was for medicinal purposes (100 %), besides dried specimens trade (81 %) and ornamental trade (22 %). In past generations, Maracaípe community utilized a powder made of dried seahorses to prepare a tea, which they believed would relieve asthma, fatigue and bronchitis.

### Abundance trends and threats

**Fig. 4:**
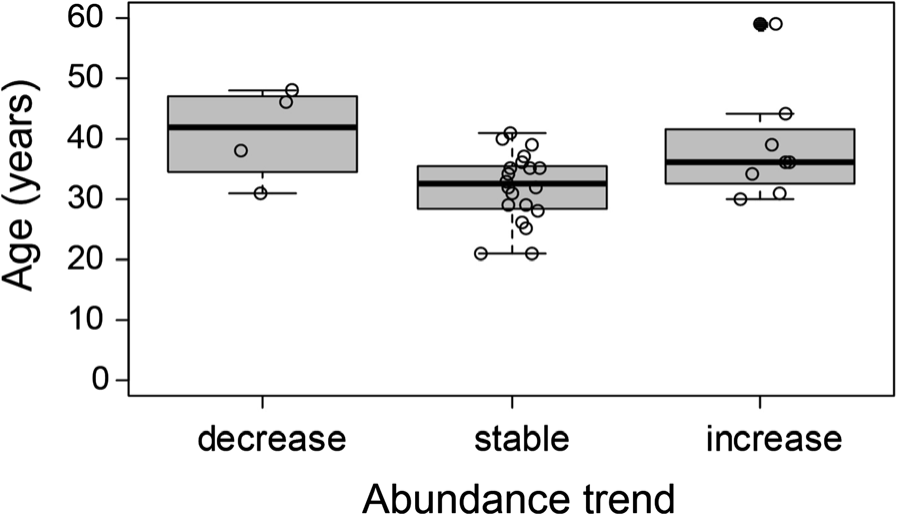
Abundance trend of seahorse population in the Maracaípe estuary according to *jangadeiros*’ knowledge and their age. The line across the box indicates the median. Dashes represent the 5th and 95th percentiles. Dashes represent the 5th and 95th percentiles, filled circles are the extreme values and open circles the raw values

**Fig. 5:**
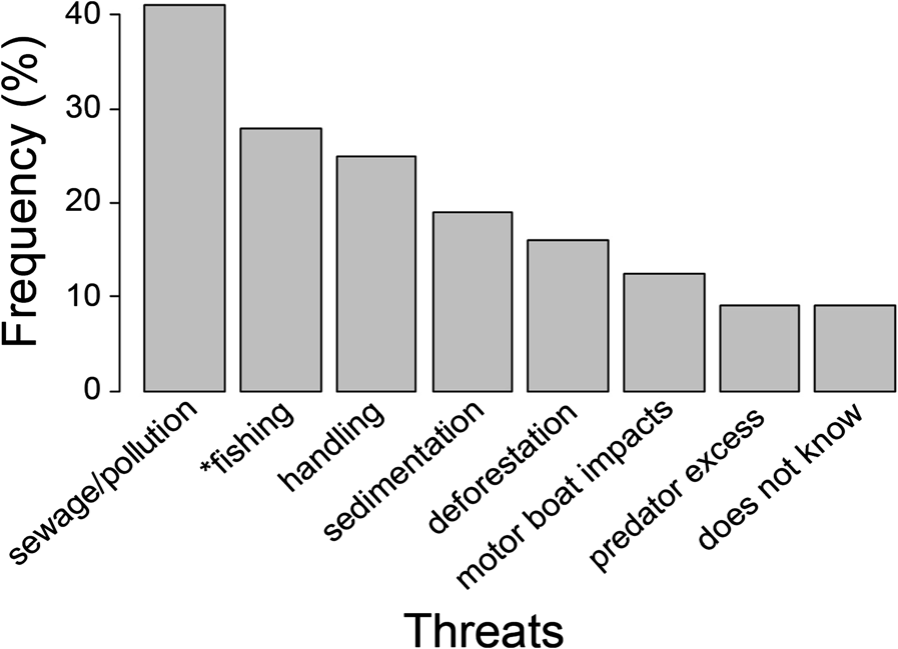
Threats to seahorses in the Maracaípe estuary according to the perception of *jangadeiros*. * seahorse collection and blast fishing

A decrease in seahorse abundance was mentioned by *jangadeiros* with a higher mean age (41 ± 3.2 years ± SE) and years of experience on seahorse-watching (11.2 ± 2.4), representing 12.5 % of the interviewees. Twenty-two percent reported an increase, and 65.5 % perceived no change in seahorse abundance since they began the touristic activity. However, we did not verify significant differences in perceptions of abundance trends (decrease, increase, no change) and the *jangadeiros’* age (Kruskal-Wallis test × ^2^ = 5.66, *p* = 0.06; Fig. 4). Among threats (Fig. 5), the main mentioned were human-related impacts, such as, sewage and pollution (41 %) and fishing activities (28 %, related to seahorse collection and blast fishing). Seahorse handling even if performed by *jangadeiros* during seahorse-watching was considered a threat (25 %), besides sand sedimentation inside the mangrove area (19 %) and its deforestation by disordered urban growth (16 %), motor boat impacts inside the estuary (12.5 %) and excess of natural predators (9 %).

## Discussion

The “Proposal of Plan for the management and sustainable use of seahorses in Brazil” [13] stated (with no previous studies) that *jangadeiros* have no knowledge of seahorse biology, but our results revealed the opposite. Maracaípe *jangadeiros* exhibited a broad knowledge-base, summarized in Table 3. By passing on this information, they can create awareness on the fragility of seahorses among visitors and mangrove users. According to *jangadeiros*, this knowledge was partially acquired from educational training promoted by non-governmental organizations and by the government environmental agency in 2007 and 2009. However, the empirical knowledge of each informant was expressed through remarks on spatial and temporal distribution related to environmental parameters, trophic ecology and reproductive aspects, such as, behavior and breeding season.

**Table 3:** Consensual responses from the informants (*n* = 32) about seahorse biological and ecological issues

Aspect	Number of different responses	Consensual responses	C.I.	Fidelity level (%)	Correspondence with scientific data
Taxonomy					
No. of species	4	One species: Longsnout (*H. Reidi*)	20	62.5	Yes [20]
Classification criteria	2	Body shape (not coloration)	31	97	Yes [24]
Distribution					
Habitat	1	Mangrove and reef	32	100	Yes [20]
Vertical distribution	3	No specific depth. (daily intense tidal depth variations in the estuary)	26	81	Yes [2]
Occurrence determinant	1	Determined by the presence of mangrove roots as anchoring points and shelter	32	100	Yes [30]
Exposition to water currents	2	Preferentially inhabit backwater areas protected from strong currents	31	97	Yes [3, 30]
Salinity	1	In the mangrove, are abundant in sites with high salinity levels	32	100	Yes [3, 35]
Water transparency	1	Abundant in high transparency level conditions	32	100	No data
Seasonal abundance	2	Abundant in summer (dry season), scarce in winter (wet season).	31	97	Yes [3]
Migration period	2	Rainy season, in winter (May to September)	32	100	Yes [3]
Reason for migration	2	To avoid low salinity levels caused by the rainy season’s freshwater input. Also are removed out of the mangrove to the sea by floods and currents	32	100	Yes [3]
Anchoring points	6	Mangrove roots	32	100	Yes [30, 33, 44]
Trophic ecology					
Diet	7	Shrimp larvae	32	100	Yes [3, 37, 38]
		Fish larvae	25	78	
Feeding behavior	4	Sucks the prey	31	97	Yes [2, 36]
Predators	10	Crabs and fishes	32	100	Yes [3, 40, 44]
Is camouflage related to color shift?	2	Yes. Is capable of changing its own color	30	94	Yes [24]
Reproduction					
Sexual dimorphism	1	Brooding pouch present on males	32	100	Yes [25]
Reproductive behavior	2	Contraction to give birth, releasing neonates as a “dust cloud”.	20	62.5	Yes [45]
Brood size	3	500 to 1000 newborns	29	91	Yes [2]
Survival rate	3	Up to 15 %	29	91	Yes [24]
Reproductive period	1	Throughout the year	32	100	Yes [3, 20, 33]

CI = number of informants who quoted a consensual response

### Socioeconomic profile

Seahorse-watching has been performed by men, mostly former fishers, with low education level. The relatively long time in the current occupation, averaging 9 years, reflects the economic dependence and suggests this touristic activity has remained profitable over the years. Management strategies to ensure the existence of seahorses should consider the socioeconomic needs of dependent users [23], such as Project Seahorse approach towards seahorse fishers in the Philippines. Seahorse-watching in Maracaípe has high socioeconomic relevance, being the primary income source for all respondents, and accounting for over 50 % of the income of those *jangadeiros* who had complementary activities [12]. Our informants’ social organization into an Association of *Jangadeiros* reflects the valorization of their image, reinforcing their cultural identity and their importance as a professional group that should be included in the discussions on sustainable use of seahorses, as they are willing to it and are also concerned about the species conservation.

### Local Ecological Knowledge on Seahorses

#### Ethnotaxonomy

The species naming by interviewees has been influenced by scientific taxonomy, acquired through educational training. The names “*cavalo-marinho do focinho longo*” (in English: “longsnout seahorse”) and “Reidi” are allusive to scientific nomenclature and agree with biological data, confirming *H. reidi* occurrence in the Maracaípe mangrove [20]. The respondents who quoted “Erecto” or “*cavalo-marinho do focinho curto*” (in English: “shortsnout”) referred to *H. erectus*, another species popularly known as shortsnout seahorse, exhibiting a robust body and a shorter snout [8]. However, there are no records of *H. erectus* in Maracaípe. Its occurrence in Brazil seems to concentrate in the southeastern and southern coast, often caught incidentally by trawl fishing, at depths of around 50 m [8]. The ethnospecies description for ‘true-seahorse’ (*Cavalo-Marinho Verdadeiro*) given by our informants match taxonomic characteristics of *H. erectus* and *H. patagonicus*.

Regarding the other ethnospecies descriptions: leafy-seahorse, king-seahorse and true-seahorse (Table 2), it should be considered that within the same species and populations, seahorses exhibit different body texture and skin filaments to simulate algae and elements of habitat structure [24, 25]. These characters, in addition to their ability to change color, confound and hinder their identification. Although scientific field research in Maracaípe had only registered *H. reidi* [20], the ecological foundations of such ethno-differentiation should be further investigated before any conclusion.

According to the scientific taxonomic criteria [24], most of our informants classify seahorses by morphological parameters, knowing that each individual can change its own coloration pattern. Perhaps the only respondent classifying seahorses by color did not attend the full educational training on seahorse biology. Maybe he participated in all stages of training, but had not absorbed certain scientific knowledge, or preferred to keep his empirical knowledge as reference. In certain communities, the meanings empirically acquired are not always replaced by exogenous knowledge presented by scientists and other professionals, who do not belong to the local reality [26]. Furthermore, ethnoecological research should not dismiss less-consensual understanding in investigated communities, as these may offer creative contrasts by eliciting original ecological patterns or revealing alternative modes of interaction between humans and other fish species [27, 28].

### Distribution

Biological data simply confirm the occurrence of seahorses in Maracaípe mangrove and adjacent reefs [20]. Informants added that inside the local estuary, seahorse habitat preference is driven by habitat structural complexity instead of depth, in agreement with literature [2, 29, 30]. In Maracaípe, the shallow estuarine area suffers intense daily tidal depth variations and thus the seahorse’s vertical movement follows these natural dynamics. Mangrove structures (e.g. roots, fallen wooden branches) provide a complex habitat with plenty of holdfasts, shelter and feeding opportunities, which are determinant factors for seahorse occurrence and habitat selection on our study site.

Seahorses use diverse natural and artificial holdfasts, demonstrating their adaptive ability, although often exposing themselves to the risk of human exploitation [31, 32]. Mangroves play an important role as habitats for seahorses during all their life stages [29, 30, 33]. Thus, seahorses can be used as flagship species for coastal zone protection [34], highlighting the need for mangrove conservation, which can benefit several other species.

Salinity and water transparency decrease concomitantly during the rainy season (austral winter) at Maracaípe estuary [19, 20], coinciding with the migration period reported by *jangadeiros*. However, poor water visibility could distort this perception, according to a respondent who stated that seahorses remain in the mangrove during winter, but are not seen because of water turbidity. Additionally, greater seahorse abundance in clear waters may be biased by conditions of better visibility during dry season. Seahorses may have seasonal migrations, although little is known about this movement pattern [2, 25]. Fishers along northeastern and northern Brazil have also reported seasonal seahorse migrations to saltier waters in the austral winter months [3]. Although the minimum salinity recorded for the Maracaípe estuary in the rainy season was 1 ppm [19], seahorses were found in points with salinity limits from 5 to 40 ppm, focusing on locations with an average of 26 ppm [20]. Euryhaline fishes inhabiting estuarine areas, such as *H. reidi,* are able to withstand salinity changes [2]. However, in some cases they might not survive the extreme variation caused by the rain freshwater inflow and flooding rivers [35].

Maracaípe informants reinforced the ethnobiological evidences of seasonal migrations possibly being related to abrupt changes in salinity. This potential migratory pattern should be further investigated to understand seahorse population dynamics and to implement more contextualized conservation measures that consider local variations in habitat use by *H. reidi* when designing marine protected areas [3, 25].

### Trophic ecology

The consensual informants’ description follows scientific research. Seahorses are indeed generalists and voracious ambush predators, feeding on live moving preys [2, 36], from zooplankton to small fishes, especially microcrustaceans [37, 38]. The “mud worms” quoted by *jangadeiros* are possibly nematodes, polychaetes and oligochaetes. These food items were found in *H. reidi* stomach contents in the Mamanguape estuary, northeastern Brazil [37].

There are no biological data considering *Sargassum* spp. on seahorse diet, although several *jangadeiros* mentioned it. We believe our informants made this trophic association because they often see seahorses drifting attached to these algae. In fact, drifting on *Sargassum* spp. is one of the ways by which seahorses reach inside the estuary in strong current events, as reported by *jangadeiros*, and this corroborates dispersal and movement pattern studies on other *Hippocampus* species [39–41]. Respondents may have quoted sludge as a food item on seeing seahorses foraging in the sediment or inspecting the substrate, probably seeking prey, similar to the behavior described for *H. reidi* in a natural environment [2, 42]*.*

There is evidence that Syngnathidae are not targeted by specialized predators, but by generalist or opportunistic species [40], such as the fishes quoted as seahorse predators in the Maracaípe estuary and also reported by seahorse fishers along Brazilian coast [3]. Partial predation by crabs on seahorses have also been reported by biological and ethnoecological studies (e.g. [3, 43, 44]). This interaction is probably an agonistic behavior, as both species share the same habitat [3].

### Reproductive aspects

Despite the seahorses’ unusual reproductive characteristics [25], *jangadeiros* showed remarkable knowledge, corroborating the scientific data. Our respondents accurately recognized seahorse’s sexual dimorphism and brood size, information reportedly acquired through educational training. This shows that empirical knowledge alone would not allow for an accurate conclusion about seahorse sexual dimorphism.

At birth, the male performs muscle contractions to expel the offspring (resembling miniatures of the adults) out of the brood pouch [45]. Such behavior was observed by most *jangadeiros* when displaying seahorses to tourists in glass containers. Thus, males in advanced pregnancy stages are captured, posing potential harm to this *H. reidi* population if the handling stress significantly interferes with reproductive success. Studies demonstrate that stress affects fish reproduction (negatively or positively) in many ways depending on the nature and severity of the stressors [46, 47]. Therefore, we highly recommend further studies on the effects of such touristic activities on seahorse reproduction. Knowledge of how these stressors affect the physiology of fish species can inform critical conservation strategies [47].

Studies have reported brood size of seahorse species ranging from 100 to approximately 2000 newborns [2], closely matching the information provided by *jangadeiros*. The informants acknowledged that seahorses release many neonates at each reproductive event, but recognized that only few reach the adult phase due to high predation in early life stages, befitting biological data [24]. However, there are no accurate values for seahorse survival rate, because it is difficult to assess its initial planktonic phase [25]. For conservation purposes, it is an important fact that the informants acknowledged these aspects of the species survival fragility.

The reproductive period mentioned by the informants agrees with biological data (*e.g.,* [20, 33, 44, 48]) and with perception of fishers from north and northeastern regions of Brazil [3] (Table 4). The temporal variation in different sites suggests the reproductive peak is linked to site-specific environmental conditions [24, 48]. In Maracaípe, the intensification of reproduction in the rainy season could be related to the increased strength of currents and floods, facilitating the dispersion of newborns in the planktonic phase out of the estuary. It could also be related to the increased water turbidity, hindering the action of predators on *H. reidi* and reducing *jangadeiros’* catch pressure.

**Table 4:** Data on *H. reidi* reproductive peak, *in situ*, along Brazilian coast

Author	Site	Months of reproductive peak	Season	Rainfall pattern	Data source
[3] Rosa *et al.*, 2005	Brazil (N and NE)	–	Winter Summer	–	Ethnobiological
[20] Silveira, 2005	Maracaípe, Penambuco (NE)	June to october	Winter	Rainy season	Biological
[44] Rosa *et al.*, 2007	Brazil (NE, SE and S)	October to february	Summer	–	Biological
[33] Osório, 2008	Pacoti and Malcozinhado estuary, Ceará (NE)	January to march	Summer	Rainy season	Biological
[48] Mai; Velasco, 2012	Delta do Parnaíba, Piauí (NE)	May to november	Winter	Dry season	Biological
Present study	Maracaípe, Pernambuco (NE)	May to september	Winter	Rainy season	Ethnobiological

On another estuary of northeastern Brazil, it was suggested that *H. reidi* prefers conditions of higher salinity and better visibility of the dry season to intensify courtship rituals, as a larger number of egg-bearing males were observed in the beginning of the rainy season [33]. The author raised a hypothesis that newborns and juveniles could benefit from a greater supply of nutrients in the estuary during the rainy season. Nevertheless, the decrease in salinity levels in the rainy season could be a limiting factor for survival, according to the juvenile mortality reported in Maracaípe [20]. The consensual response among *jangadeiros* was that, in winter, the seahorses in the mangrove move to the sea. The information given in the reproductive biology and the influence of environmental variables should be further investigated. Contradictions between LEK and biological data can elucidate facts not yet perceived and suggest new investigative approaches and working hypothesis [49, 50].

### Human uses of seahorses

The medicinal use of seahorses as described by the interviewees is documented in several regions of Brazil and Latin America [51, 52]. Nevertheless, *jangadeiros* argued not to believe in its effectiveness and they consider it an obsolete and unnecessary practice, because today there are drugs available for such diseases. This perception is positive for conservation and reduces fishing pressure for medicinal purposes. Thus, the respondents have no involvement with seahorse fishing or trade, contrasting to other Brazilian communities [3]. However, seahorses are still overexploited in Brazil for ornamental trade [53].

### Abundance trends and threats

Older fishers generally have the longest baseline of changes in the abundance of marine organisms [54, 55]. However, seahorse-watching in Maracaípe is a relatively recent activity. Thus, possibly the *jangadeiros* still do not perceive short-term changes in the abundance of seahorses or such changes have not occurred. In this context, we expected that perceptions of changes in abundance would not vary according to the stakeholder’s age. Nevertheless, despite the non-significant statistically tested result, a decrease in abundance was mentioned by four older informants. Thus, the hypothesis of long-term declines in abundance should not be dismissed.

The informants reported mostly human-related impacts encompassing main global threats to seahorses, such as, fishing, habitat loss, collecting for ornamental and dried specimen trade [6, 24]. By considering the handling of seahorse specimens as a threat, the *jangadeiros* were concerned about the sustainability of their economic activity and assumed that it could have a negative impact. Along with pollution and sewage discharge, motor boat chemical impacts (fuel, oil, petroleum products, solvents, paint and resins waste from boat repair) can potentially be detrimental to marine habitats [56, 57] and also to seahorses, once they bioaccumulate xenobiotics [58].

Regarding habitat loss, in Maracaípe it is specially driven by illegal blast fishing, deforestation and anthropization of mangrove areas, besides sedimentation (referred to sand deposition upon the muddy sediment). According to *jangadeiros*, the geological phenomenon of sand deposition in the estuarine area of Maracaípe river has drastically increased in recent years, covering the submersed roots of mangrove vegetation and thus decreasing the availability of holdfasts for seahorses. Sites where the original sediment was mangrove mud were covered by sand, decreasing the river channel depth and hindering navigation in some points (informants’ comments by January 2016). This perceived increase in sand sedimentation could be related to the intensification of deforestation [59].

The environmental human-related pressures reported by *jangadeiros* for the Maracaípe mangrove were also registered in another study [59], where the overall index of impact on the Maracaípe estuary was considered high, caused by the lack of urban planning and disregard for environmental laws. Part of these alterations were linked to the uncontrolled growth of tourism and the Industrial Port of Suape, one of the main economic centers of Brazil, located 23 km away from the study site [59].

Although anthropogenic action and environmental changes represent a threat to marine biodiversity, fish populations can also be affected by unrevealed natural cycles [5]. Hence, for conservation assessments, seahorse populational trends in Maracaípe need to be investigated considering: a) the fact that seahorses exhibit natural population variations even in the absence of human exploitation [60, 61]; b) considering the predominating complexity of anthropogenic interactions at Macaraípe estuary, *i.e.,* given the existence of other major potential driving causes of a decline (*e.g*., Fig. 5), and c) the hypothesis of insignificance of handling impacts by seahorse-watching operators in a given population health. Given the Data Deficient conservation status of many seahorse species, including *H. reidi*, there is an urgent need for more research, such as long-term monitoring of wild populations [5].

## Conclusion

The informants presented a broad knowledge, acquired through empirical experience and knowledge-exchange with scientists and marine conservation practitioners. The association between these different sources of knowledge complemented one another. Despite the gaps remaining on biological data about certain aspects of seahorse biology, the respondents provided reliable information on all questions, adding ethnoecological remarks not yet assessed by conventional scientific surveys. They report a seasonal seahorse distribution that deserves further investigation, suggesting a migratory pattern for the local population between the mangrove and coastal reefs.

The economic dependence of Maracaípe *jangadeiros* on seahorses led them to find it better to keep seahorses alive *in situ* as a touristic attraction rather than fishing them for ornamental and dried trade, or personal medicinal use. If cautiously guided, Maracaípe *jandeiro*’s approach could potentially be used for the species conservation once the stakeholders agree to it. However, understanding the impact of this tourist activity on seahorse populations is paramount to contribute to its sustainable trajectory.

Finally, we suggest the Brazilian proposal of management plan for sustainable use of seahorses be revisited: a) focusing also on the non-extractive use of seahorses, delineating proper conservation strategies and monitoring seahorse-watching activities, b) taking into consideration the knowledge of Maracaípe *jangadeiros* reported herein and recruiting these stakeholders’ participation in the resource management. We believe stakeholders who are knowledgeable about seahorse biology and threats, can assist and be more compliant with management measures and are more likely to adopt practices for a sustainable use of seahorses.
